# Numerical Model for Determining the Magnetic Loss of Magnetic Fluids

**DOI:** 10.3390/ma12040591

**Published:** 2019-02-16

**Authors:** Miloš Beković, Mislav Trbušić, Sašo Gyergyek, Mladen Trlep, Marko Jesenik, Peter S. B. Szabo, Anton Hamler

**Affiliations:** 1 Faculty of Electrical Engineering and Computer Science, University of Maribor, 2000 Maribor, Slovenia; mislav.trbusic@um.si (M.T.); mladen.trlep@um.si (M.T.); marko.jesenik@um.si (M.J.); anton.hamler@um.si (A.H.); 2 Jozef Stefan Institute, 1000 Ljubljana, Slovenia; saso.gyergyek@ijs.si; 3 School of Engineering and Physical Science, Heriot-Watt University, Riccarton, Edinburgh EH14 4AS, UK; P.Szabo@hw.ac.uk

**Keywords:** magnetic fluid, loss model, specific absorption rate, linear response theory

## Abstract

Magnetic fluid hyperthermia (MFH) is a medical treatment where the temperature in the tissue is increased locally by means of heated magnetic fluid in an alternating magnetic field. In recent years, it has been the subject of a lot of research in the field of Materials, as well as in the field of clinical testing on mice and rats. Magnetic fluid manufacturers aim to achieve three objectives; high heating capacity, biocompatibility and self-regulatory temperature effect. High heating power presents the conversion of magnetic field energy into temperature increase where it is challenging to achieve the desired therapeutic effects in terms of elevated temperature with the smallest possible amount of used material. In order to carry out the therapy, it is primarily necessary to create a fluid and perform calorimetric measurement for determining the Specific Absorption Rate (SAR) or heating power for given parameters of the magnetic field. The article presents a model based on a linear response theory for the calculation of magnetic losses and, consequently, the SAR parameters are based on the physical parameters of the liquid. The calculation model is also validated by calorimetric measurements for various amplitudes, frequencies and shapes of the magnetic field. Such a model can serve to help magnetic fluid developers in the development phase for an approximate assessment of the heating power.

## 1. Introduction

Magnetic fluids attract attention because of their unique properties, which, in several respects, differ considerably from those of bulk materials. A great deal of promising applications have arisen because of this, especially in medicine for cancer treatment using hyperthermia. This is a treatment where magnetic-fluid tumour-loaded tissue is exposed to an AC magnetic field which, in the case of mild hyperthermia, results in a temperature rise from 41–43 °C. An ideal hyperthermia treatment should destroy the tumour cells selectively without damaging the surrounding healthy tissue [1].

The heating effect of a magnetic fluid, when exposed to an AC magnetic field, is a direct consequence of different physical mechanisms. The transformation of field energy into heat depends strongly on the frequency and amplitude of the AC field. It also depends on the nature of the particles, such as particle size, surface modification, and the carrier liquid. These arguments have been dealt with others in works [1,2,3,4,5,6,7,8,9,10]. For the successful use of fluids for medical purposes, knowledge of heating power and its temperature dependence is essential.

This work represents a systematic study of the heating effect of superparamagnetic nanoparticles in alternating (amf) and rotating magnetic field (rmf). Results of analytical calculations are compared with calorimetric measured results for various frequencies and amplitudes of the magnetic field for the purpose of hyperthermia treatment.

## 2. Model of Magnetic Losses Calculation

The magnetic relaxation time corresponding to the time in which the direction of the magnetic moment of nanoparticle flip and reverse its direction under the influence of external magnetic field, representing a crucial parameter that defines the power dissipation.

The two mechanisms of alignments mentioned above indicate that, in the theory of magnetic nanoparticles relaxation, there are two possible relaxations; the first one is called Néel relaxation (*τ*_N_) and the second is Brownian relaxation (*τ*_B_).

Néel relaxation is dominant for smaller particles, and magnetic moments follow the alternating magnetic field by rotating against the anisotropic energy barrier within the particles. Brownian relaxation, which dominates larger particles, is the action of whole-scale rotation of particles within the base fluid. The expressions for *τ*_N_ and *τ*_B_ are given by Equations (1) and (2), were τ_0_ is an exponential factor 1 × 10^−9^ provided by McNab et al. in Reference [11] and later Rosensweig [12], *K*_a_ is the anisotropy constant in J/m^3^, *V*_p_ is the volume of particle in m^3^, k_B_ is a Boltzmann constant (1.38 × 10^−23^ J K^−1^). *T* is an absolute temperature in K, *V*_h_ is the hydrodynamic volume of spherical particles, including surfactant thickness, and *η* is the viscosity of the base fluid.

(1) $$\tau_{N}=\tau_{0}e^{\frac{K_{a}V_{p}}{k_{B}T}}$$

(2) $$\tau_{B}=\frac{3V_{h}\eta }{k_{B}T}$$

For determining magnetic losses, it is not important which of the two relaxation mechanisms will prevail, but the resulting relaxation time *τ*, which is calculated according to Equation (3)

(3) $$\tau^{-1}=\tau_{B}^{-1}+\tau_{N}^{-1}$$

The model of magnetic fluid losses calculation is set for the rotating magnetic field, where the orthogonal breakdown of magnetic variables implies two main directions, *x* and *y*. According to P. Cantillon-Murphy [13] a rotational magnetic field excitation has two complex components ${\hat{h}}_{x}=H_{ex}$ and ${\hat{h}}_{y}=jH_{ey}$, and, therefore, components of magnetization vector *M* can be calculated as

(4) $${\hat{M}}_{x}=\chi_{x}\frac{{\hat{h}}_{x}}{j\omega \tau +1},\;{\hat{M}}_{y}=\chi_{y}\frac{{\hat{h}}_{y}}{j\omega \tau +1}$$

In above equation *ω* is the magnetic field frequency (2π*f*) and *χ* is the maximum value of a chord susceptibility, calculated from a maximum value of time changing magnetic field in the case of an alternating field, or its amplitude in the case of a rotating field. Both cases can be calculated by Reference [13].

(5) $$\chi_{x}=\frac{\varphi M_{d}}{H_{ex}}\left(\coth\left(\alpha_{x}\right)-\frac{1}{\alpha_{x}}\right),\;\chi_{y}=\frac{\varphi M_{d}}{H_{\mathrm{ey}}}\left(\coth\left(\alpha_{y}\right)-\frac{1}{\alpha_{y}}\right)$$

The unitless Langevin parameter α can be calculated as α = µ_0_ *V*_p_ *M*_d_ *H*_e_/k_B_*T*, in the same manner for *x* and *y,* replacing *H*_e_ with a field excitation vector component, *φ* is the volume fraction of magnetic nanoparticles in suspension, and *M*_d_ is the bulk saturation magnetization. The time-averaged volumetric heating power *P* in W/m^3^ can be calculated with Equation (6), and consists of two components of a rotating field, whereas, in the case of an alternating field, only one component remains.

(6) P=µ02Re(jωM^xh^x*+jωM^yh^y*)

Here, Re stands for a real part of the complex value, ${{\hat{h}}_{x}}^{*}$ and ${{\hat{h}}_{y}}^{*}$ denote a complex conjugation of ${\hat{h}}_{x}$ and ${\hat{h}}_{y}$, respectively. The Specific Absorption Rate (SAR) [8] is the most commonly used parameter to compare different materials in similar magnetic conditions, or vice versa. It is also the most common way to determine heating power experimentally, and the aim of this paper is to calculate SAR and compare it to measured values; it is evaluated using Equation (7) were *ρ*_m_ is the mass density of the magnetic nanoparticles.

(7) $$\mathrm{SAR}=\frac{P}{\rho_{m}}$$

## 3. Materials and Methods

### 3.1. A: Characterization of A Magnetic Fluid Sample

For evaluating the loss model, we used a commercially available sample of magnetic fluid that had previously been exposed to SAR characterization for alternating and rotating magnetic fields. Maghemite nanoparticles (γ-Fe_2_O_3_) dispersed in mineral oil form a stable suspension with a saturation magnetization of 42.3 kA/m; when divided with a bulk saturation magnetization of 400 kA/m it reveals the volume fraction of magnetic nanoparticles *φ* = 10.57%.

**Figure 1:**
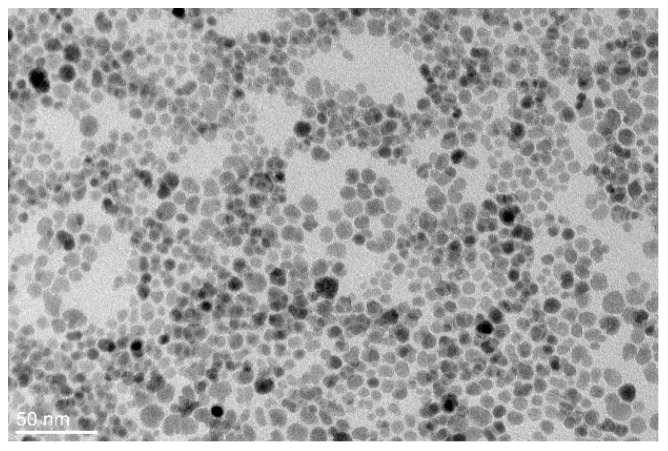
TEM image of maghemite nanoparticles in a magnetic fluid sample.

A Transmission Electron Microscopy (TEM) analysis was performed and the resulting image of ferrofluid nanoparticles is shown in Figure 1. Usually fatty acids are used for colloidal stabilization of magnetic nanoparticles in non-polar hydrocarbons. Such capping agents are approx. 1 nm in length [14], increasing the hydrodynamic radius of the nanoparticles for the same value. For this reason, we have used the same value for the estimation of the relaxation time. 

**Figure 2:**
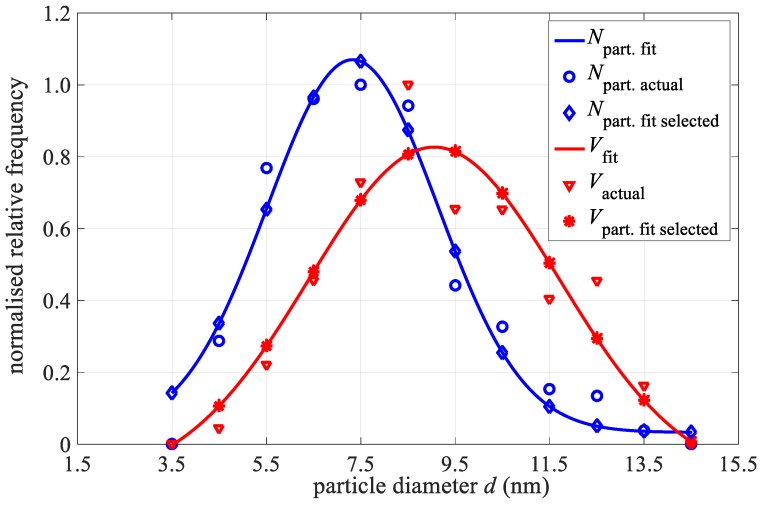
TEM images of the nanoparticles and the corresponding nanoparticle empirical size distribution (solid symbols) fitted with Gaussian curves (lines).

Empirical, number-weighted, particle-size-distribution functions were estimated from the TEM images, resulting in the distribution, seen in Figure 2 (blue circle, *N*_particle actual_). The particle size is given as an equivalent diameter—the diameter of a circle having the same surface area as the imaged particle. At least 500 particles were measured. The empirical number-weighted distribution functions were normalized and fitted with a log-normal distribution function. The average size (*d*_NTEM_) and standard deviation (*σ*_NTEM_) were calculated from known relationships between the arithmetic moments and the parameters of the log-normal distribution function denoted as *N*_part. fit_ [12]. The diamond marker on this curve indicates values for selected diameters ranging from 3.5 to 14.5 nm in a 1 nm step. To obtain the volume-weighted particle-size-distribution functions the empirical number-weighted distributions were transformed to volume-weighted ones, normalized and fitted with log-normal distribution functions. The parameters *d*_VTEM_ and *σ*_VTEM_ were obtained in the same fashion as the number-weighted particle-size-distribution parameters [15]. Normalised actual values of particles volume are marked with red triangles, whilst the Gaussian fit curve is a red-star curve. At some values, a huge difference of up to 20% is seen from the fit curve and actual values, hence, the latter were selected for further analysis.

The result of the TEM analysis is the vector of the volume fraction of each size-group of particles *V*_actual_. In order to use this result, we had to divide it with the vector sum, and multiplied it with the actual volume fraction of magnetic nanoparticles (8).

(8) $$\varphi_{d}=\frac{V_{\mathrm{actual}}\;\varphi }{\sum_{d_{\min}}^{d_{\max}}V_{\mathrm{actual}\_d}}\cdot 100\%$$

**Figure 3:**
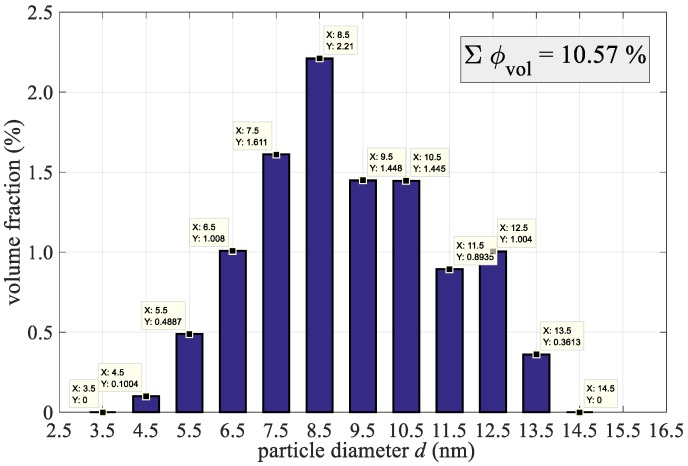
Volume percent of each size-group of particles in suspension.

The result is the volume percent of each size-group of particles in suspension, as can be seen in Figure 3. For example, the volume fraction of a 7.5 nm size-group is 1.611%, whilst the sum of all particles is 10.57%, as previously calculated from measurements. 

### 3.2. B: Measurements of Magnetic Fluid Losses

To evaluate the Specific Absorption Rate (SAR) of magnetic fluid experimentally there are several methods, where calorimetric measurement is used most commonly. Because of its simplicity it is widely accepted, and SAR can be calculated using Equation (9), where *c* is specific heat capacity, *ρ* is the density of the sample and *m*_Fe_ is the mass of magnetic nanoparticles per unit volume. The ratio d*T*/d*t* is the time derivative of measured temperature, where its maximal value is relevant for SAR evaluation; usually it occurs within the first few seconds of magnetic field switch-on. Temperature measurement is realised with a FISO fiber optic sensor.

(9) $$\mathrm{SAR}=\frac{c\;\rho }{m_{\mathrm{Fe}}}{\left(\frac{dT}{dt}\right)}_{\max}$$

We have developed two systems for experimental evaluation of SAR, the first one for an alternating magnetic field, and the second for a rotating magnetic field. System properties and methods are presented in Reference [3] for the alternating and Reference [4] for the rotating field, where, in this paper, only the experimental results are used for comparison with the calculation model. 

## 4. Results and Discussion

The calculation of the heating power or the SAR of the magnetic fluid was divided into individual size groups, as evaluated in Figure 3. We divided the liquid artificially into twelve parts, calculated their individual contribution to the total SAR losses, and summarized them at the end. According to our problem, Equation (6) can be written as follows.

(10) P=µ02Re(∑i=112(jωM^xih^x*+jωM^yih^y*))

**Figure 4:**
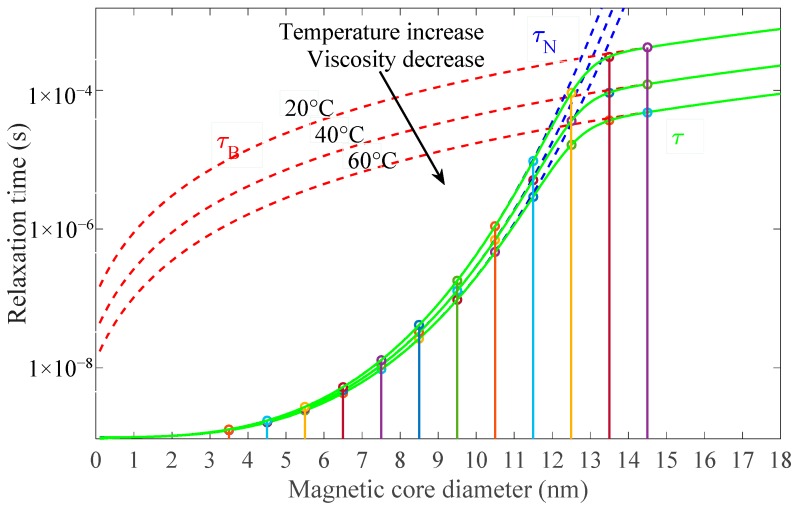
Calculated relaxation times for different magnetic core sizes for three temperatures and viscosities.

According to the equations of the model, it is first necessary to determine the relaxation times for each individual size group of particles with respect to Equations (1), (2) and (3); for the SAR calculation, the resulting relaxation time *τ* is relevant, as seen in Figure 4. 

As the faster of both mechanisms prevails, we can conclude that the Neel relaxation mechanism prevails for particles below 12 nm in diameter whilst, for larger particles, the Brownian relaxation is active. Circular markers indicate the relaxation times for actual particle sizes, and the graph shows the dynamics of the change in the case of increased temperature, and, at the same time, the lowered viscosity of the carrier fluid [16]. 

**Figure 5:**
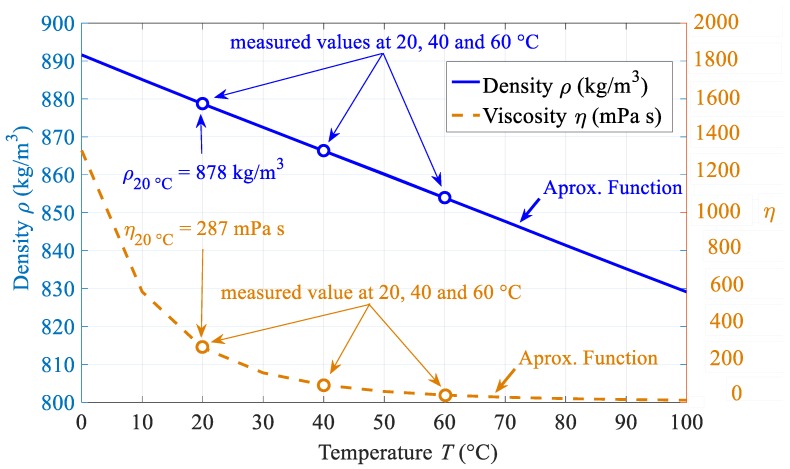
Temperature dependence of density and viscosity of mineral oil based magnetic fluid with 10.57% volume fraction of maghemite magnetic nanoparticles, three measured points and approximation function for the temperature range from 0 to 100 °C.

The necessary variables for the SAR evaluation are the density and the viscosity of the magnetic fluid. The values of both variables change significantly with temperature; hence, this must be included in the model since it may have an impact on the relaxation time calculations as seen above. For the used sample of magnetic fluid three measurement points of density and viscosity had been measured; 20, 40 and 60 °C whose values are plotted in Figure 5. In the same graph an approximation function of both variables is also plotted whilst for other temperatures proper temperature dependent curves must be measured. 

**Figure 6:**
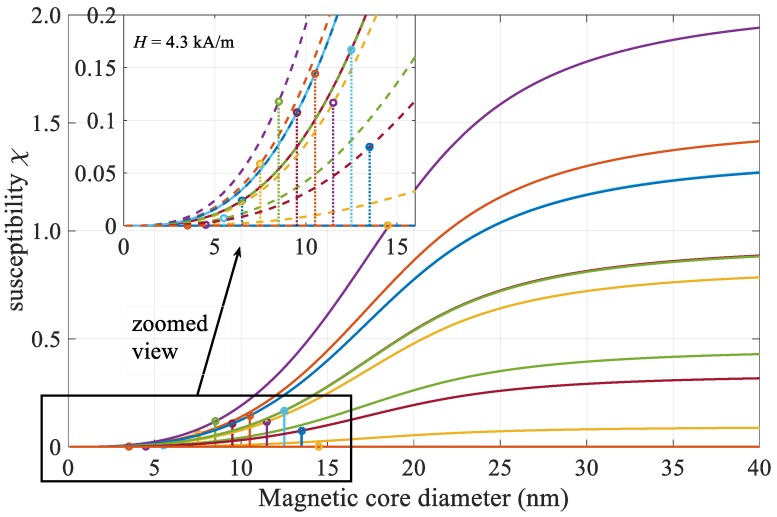
Magnetic nanoparticle chord susceptibility size dependence for magnetic field of 4.3 kA/m.

Langevin’s function (coth(*α*)-*α*^−1^), which appears in the expression for complex susceptibility (5), depends only on the volume of the particles at a constanting temperature, the external magnetic field, and the magnetization of the material. Figure 6 displays this function multiplied with weight factor (*φM*_d_/*H*_e_) according to (5) for the change of particle size from 0–40 nm, where twelve lines are plotted for selected volume concentrations from Figure 3. The zoomed view reveals the actual values of calculated susceptibilities for selected size groups, ranging from 0 to 0.17 for an applied alternating magnetic field of 4.3 kA/m.

**Figure 7:**
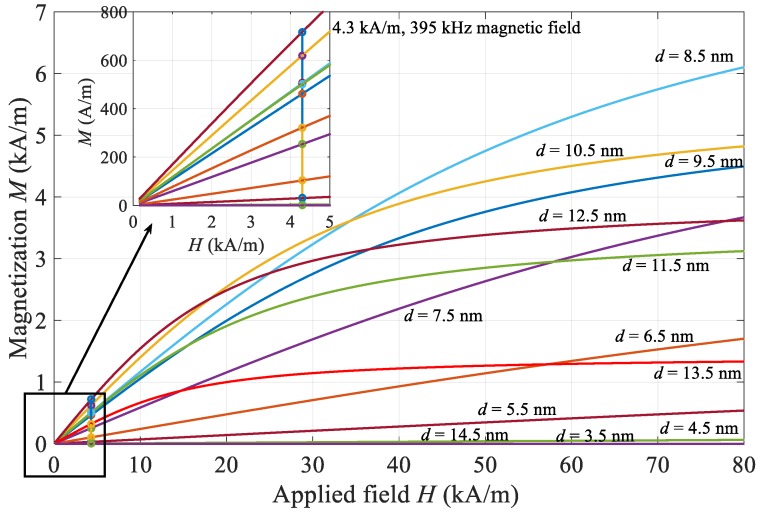
Magnetization of various size particles for a magnetic field of 4.3 kA/m and 395 kHz.

Calculated chord susceptibilities are used for the magnetization component calculation (5) as a key parameter for determining the heating power (6) of the magnetic fluid exposed to a magnetic field. Individual contributions are displayed in Figure 7 for the 4.3 kA/m, 395 kHz magnetic field and the corresponding concentrations of individual size groups. According to Reference [17] linear response theory is valid for this system, since particles are below 20 nm and magnetic fields are below 10 mT.

The following are the results of calculations for examples of alternating and rotating magnetic fields and comparison with measured values.

### 4.1. Alternating Magnetic Field

**Figure 8:**
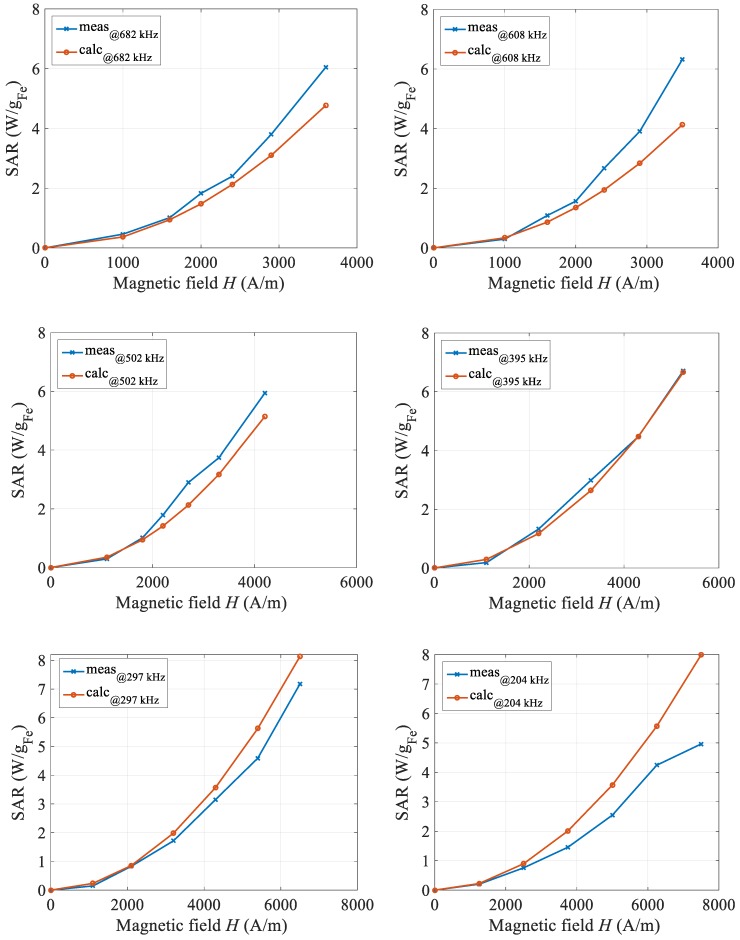
Comparison of measured and calculated SAR curves for six different frequencies of the magnetic field.

The comparison between calorimetric measured and calculated values of magnetic fluid heating SAR, obtained at the same frequencies are shown in Figure 8. For six selected frequencies, the calorimetric measurements were made in the same way as described in Reference [3] and comparing them with the above-described SAR calculations.

The next series of figures revealed the best match between calculated and measured SAR values at the field frequency of 395 kHz. When the field frequency is higher, the measured SAR values are higher, while at lower values the reverse is true. The deviations are in the range of ±15 percentage, and it should be noted that, even in the measured values, a certain degree of a certain degree of error exists, due mainly to the determination of the maximal temperature derivative. From this, we can conclude that the proposed calculation describes well the values of the SAR parameter in the selected frequency and amplitude of magnetic field strength range with a certain degree of tolerance.

### 4.2. Rotating Magnetic Field

The model for magnetic losses indicates higher values of SAR in the case of the rotating magnetic field seen in Equation (6), where two contributions, *h_x_* and *h_y_*_,_ contribute equally to total losses. This theoretical assumption has been validated experimentally, and results are summed in Reference [4], explaining the experiment and method in details. For calculating the rotational losses, we extended the model of the calculation to two components of the magnetic field, as indicated by (1)–(7). 

**Figure 9:**
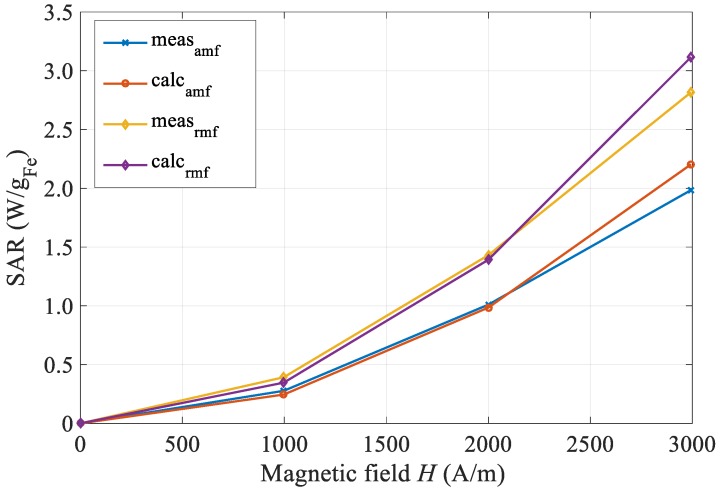
Comparison of measured and calculated SAR curves for alternating and rotating magnetic field at 402 kHz.

We tested the loss model with experimental results for one frequency of 402 kHz. In the measuring system [4], we performed three calorimetric measurements for the single-alternating magnetic field at amplitudes 1, 2 and 3 kA/m, and double (*x* and *y*) system power supply at the same field amplitudes for achieving the rotational magnetic field. Expectedly, the fluid was heated more intensely at a rotating magnetic field by 40 to 50%, resulting in the same ratio of SAR losses. Both the measured curves, the alternating and the rotational fields, are shown in Figure 9 for the same magnetic conditions (amplitude, frequency and type of magnetic field). We also performed a calculation of SAR magnetic losses, whereby, in the case of a rotational field, we take into account the complex value of the contributions in *x* and *y* magnetic field components in such a way that the total contribution is summed up in a vector manner. The result of the analysis of the calculation is plotted in the same graph, where we see a good match between the model calculation and the actual measurements.

## 5. Conclusions

In the manufacturing process for magnetic fluids for medical hyperthermia, one of the key desires is finding the maximum heating power of the magnetic nanoparticles, and, consequently, the smaller amount of material used in treatment to achieve the desired target temperatures. Often, fluid manufacturers do not have a realistic idea of the heating power of the sample until standard calorimetric measurements of the SAR are performed. The aim of this article was to test known equations for evaluating losses, and then validate the calculation mode experimentally.

Comparison of the loss calculation model and the measurement indicates a good correlation of the results within a relatively broad frequency spectrum. Since both cases involve certain deviations, it would be worthwhile to pay more attention to the repeatability of measurements in the continuation, as well as to experiment with different samples of magnetic fluid. In the case of calculations, more attention should be paid to the measurement of the physical parameters of the liquid and, in particular, their temperature dependence, since, in most cases, they are altered significantly, even with minor temperature changes. The loss calculation model can be used as a good basis only with precise knowledge of all input parameters of the magnetic fluid. In the case of model validation in other samples, the model could be used to study different magnetic materials, carrier liquids, different particle size distributions, different concentrations, different amounts of surfactant, etc.

The comparison of magnetic fluid SAR characteristic between calculated and measured results in case of amf results reveal a good match between them. However, if we compare magnetic losses between the alternating and rotational magnetic fields, we find that the rotation field really creates greater losses, but, on the other hand, only a 50% larger loss does not outweigh the complexity of the system needed to create a rotational magnetic field of sufficiently large amplitudes. In this case, two rectangular coils are required, and, consequently, two dual phase-shifted power supplies. In the case of amf, however, the sample is in the centre of the coil where the field is strongest, and can easily reach greater amplitude, and, therefore, the alternating field remains the dominant field shape in the case of medical usage of magnetic fluid hyperthermia.
